# Left-sided diaphragmatic eventration in chronic obstructive pulmonary disease patient: a rare clinical image

**DOI:** 10.11604/pamj.2025.50.74.46778

**Published:** 2025-03-14

**Authors:** Lajwanti Laxmandas Lalwani, Priyanka Khemraj Chilhate

**Affiliations:** 1 Department of Cardiovascular and Respiratory Physiotherapy, Ravi Nair Physiotherapy College, Datta Meghe Institute of Higher Education and Research, Sawangi (Meghe), Wardha, India

**Keywords:** Diaphragmatic eventration, chronic obstructive pulmonary disease, hemidiaphragm

## Image in medicine

A diaphragmatic eventration is a congenital abnormality characterized by a failure of muscular development, either entirely or partially, in one or both hemidiaphragms. In clinical practice, eventration of the diaphragm is the unusual elevation of one-half of a structurally normal diaphragm due to paralysis, aplasia, or relatively decent at different degrees of muscle fibers. A 69-year-old male working as a farmer was referred to the respiratory medicine department with a history of cough with mucoid expectoration, off-and-on fever, chest pain, and breathlessness affecting his activities of daily living. On examination, the patient disclosed a five-year history of chronic obstructive pulmonary disease (COPD). Clinical assessment revealed decreased bilateral chest wall movement and a centrally positioned trachea. Palpation corroborated these observations. Further examination disclosed diminished left-sided chest wall excursion in the inframammary, infra-axillary, and infra-scapular regions, accompanied by reduced tactile vocal fremitus and no air entry in the left middle and lower lobes on auscultation. Bilateral decreased breath sounds and basal crackles were noted on the right side. The diagnostic imaging in a photofluorogram chest radiography (A) and computed tomography (B) imaging revealed that the left hemidiaphragm was elevated with a smoother margin than the contralateral side suggestive of diaphragmatic eventration. The fluoroscopic-guided Sniff test (C) was used to confirm the results of the imaging. He was enrolled in pulmonary rehabilitation and pharmacological therapy. Pulmonary rehabilitation helped in the reduction of dyspnoea, anxiety, and depression, increasing muscle power and endurance, and improving his quality of life.

**Figure 1:**
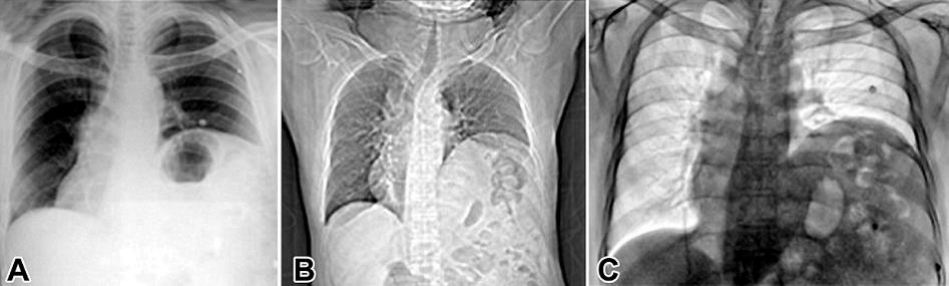
(A,B,C) left-sided diaphragmatic eventration

